# “If it’s gonnae make things better then I don’t have any issue”: Perspectives of People Who Use Drugs on the Use of Routinely-Collected Data for Research

**DOI:** 10.23889/ijpds.v11i1.3428

**Published:** 2026-06-30

**Authors:** Louise Marryat, Hazel Booth, Sarah Gray, Andrea Mohan, Senga Robertson, Sreekanth Thekkumkara, Camila Biazus-Dalcin

**Affiliations:** 1 School of Health and Wellbeing, University of Glasgow, Glasgow, UK; 2 School of Health Sciences, University of Dundee, Dundee, UK

**Keywords:** substance use, public involvement, stigma, data

## Abstract

**Introduction:**

Routinely-collected data are increasingly used to study outcomes among marginalised communities, including people who use drugs, because they capture groups often under-represented in traditional research. However, little is known about how this population feels about researchers using their data, particularly given the stigma they frequently encounter.

**Objectives:**

This public involvement and engagement study explored the views of people who use(d) drugs on the use of their routinely-collected data in research, with the aim of informing future work in this field.

**Methods:**

Participants were recruited through a recovery organisation in Southeast Scotland, UK. Two deliberative focus groups were conducted (n=11 and n=12). Each session began with a short introduction to routinely-collected data and its research uses, followed by discussion guided by a topic schedule and a creative participatory activity. Thematic analysis was carried out using NVivo v15.

**Results:**

Five overarching themes were identified. Participants expressed very low awareness that their data were being used in research and highlighted the need for clearer and more accessible transparency about data processes. Concerns about consent and personal control were common, with many wanting greater involvement or choice in how their information is used. Participants strongly supported data use when it served a clear and meaningful public benefit and when researchers were held accountable for how findings were used. Discussions also reflected worries about the accuracy, completeness, and fairness of the data held about them, particularly where misunderstandings or assumptions might be recorded. Underpinning all themes was a pervasive sense of fear and mistrust toward services that collect data, which participants felt could limit honest disclosure and ultimately affect data quality.

**Conclusions:**

People who use drugs were broadly supportive of their routinely-collected data being used in research with clear social value and transparency. However, pervasive mistrust of data-collecting services may affect data quality and should be carefully considered by researchers.

## Introduction

The use of routinely-collected data, such as hospital records and education records, has opened up a world of possibilities for researchers. This is particularly the case for researching populations who may be more challenging to identify or follow over time, such as people who use drugs, often due to stigma experienced by these groups [1]. The use of routinely-collected data not only requires the infrastructure to securely store, link and access these data, but also requires a ‘social licence’, through the support of the people whose data are used [2]. Rooney et al. (2014) described the social licence as not legally binding, but rather ‘an informal agreement that is granted by communities and relevant stakeholders to an organisation or industry working in the local area’ [2]. The lack of a social licence can have catastrophic impacts on the accessibility of routinely-collected data for research, as was seen in the ‘care.data’ fiasco in England, UK, where a lack of public education and resulting concern about the use of people’s routinely-collected data led to a public backlash [3]. In recent years substantial research has been published which examines the social licence for use of routinely-collected data in research, through perceptions of a range of publics. In general, the research suggests that, across countries, the general public are mostly supportive of sharing of routinely-collected data for research, and that this social licence holds [4, 5]. Views of specific populations mirror this, including marginalised groups such as Looked After Children [6], or people with mental health difficulties [7]. Although overall support for use of routinely-collected data was high in these groups, some concerns were raised. For example, people with mental health difficulties were less supportive of researchers accessing and using their mental health data, specifically, although support remained high overall [7].

Little is known, however, about the views of people who use/or have used drugs. This is important because people who use drugs are a particularly stigmatised group, who may reasonably have concerns about how their data are used. Routinely-collected data research requires trust that data will be used sensitively and wisely. People who use drugs frequently report being stigmatised and discriminated, often by the very services that are supposed to help them [8]. Trust may therefore be a particular barrier. In addition, some of these routinely-collected data might be particularly sensitive: this population have high levels of childhood trauma, domestic abuse, overdose, suicide, criminal justice involvement, and child removal [9–12]. People who use drugs might differ from populations previously researched in other respects, such as their demographic backgrounds. There are low levels of literacy, generally, and health literacy, in the population of people with drug and alcohol use difficulties, with reading ages reported as being 4-5 years below the level needed to read and understand standard treatment materials [13], and inadequate health literacy of 87% among this population [14]. Overall, these differences make it difficult to know whether perceptions of data use from other populations are likely to differ from those for people who used drugs. The increasing use of routinely-collected data to explore important topics such as drug-related deaths, and opioid exposure in pregnancy, requires exploration of the social licence for this particular population.

This study used deliberative focus groups, coupled with creative methods, to ascertain the views of people who use/used drugs, on the use of their routinely-collected data for research purposes. The study itself was a Public Involvement and Engagement (PIE) project, rather than research, which also generated publishable data that could be helpful to others working in this space. The aim of which was to inform both routinely-collected data research happening within the team of researchers, as well as with other researchers conducting routinely-collected data research, as well as to build relationships with the community itself.

## Methods

This PIE study, which produced publishable data, aimed to explore the views of people who use drugs about the use of their routinely-collected data (e.g. hospital records, drug and alcohol service data, social work data) for research purposes.

### Setting and Participants

Participants were recruited through a recovery organisation based in South-East Scotland in May-June 2023. The recovery organisation runs groups at three different locations: participants were recruited from two of these locations. As this was a PIE project, no specific criteria for inclusion were provided, other than that participants were attending the recovery organisation group meeting at the time of data collection, and were willing and able to participate. Due to the low levels of literacy in this population, recruitment and consent were carefully considered and discussed in partnership with the recovery organisation to achieve the right balance of informing people about their participation, and enabling a wide range of people to be included. Recruitment happened in two stages: firstly, the week before the focus groups were conducted, staff from the recovery organisation met with people who use their service and explained what the study was about, what would happen, and the voluntary nature of participation. Secondly, on the day of each group, the research team spoke with people attending the organisation’s group meeting about the study, reiterating what it would involve and the voluntary nature of participation. Those who wished to take part were then taken through the consent form verbally and paper copies were also available for each person. Their and consent was then recorded.

Participants received a £20 voucher to thank them for their time following data collection, and separately for participation in a related animation. This was lower than the recommended NIHR-rates, following discussion with staff at the recovery organisation, who were worried about risk to individuals if others knew they had accumulated higher levels of payment. The remaining money therefore was used to provide food for all attendees, regardless of participation in the study. Any surplus money went to the recovery organisation to support their group activities.

### Ethical Approval

Although this was a PIE project, due to the potential vulnerability of participants, and the plan to publish findings, ethical approval was sought and received from the University of Dundee School of Health Sciences and Dentistry ethics committee (approval number UOD-SREC-SHS-2023-015).

### Data Collection and Management

Data were collected through two deliberative focus groups. Unlike traditional focus groups, deliberative focus groups attempt to educate and inform participants about a topic prior to a more general discussion, with the aim of generating better quality data on the topic [15]. As previous research on perceptions of the use of routinely-collected data highlighted a lack of understanding about data use and linkage [5], it was decided that this approach would be most suitable. The focus groups therefore started with a short introduction, led by a researcher experienced in using these data (Marryat), describing what routinely-collected data is in Scotland (e.g. data collected by services such as schools, hospitals, drug and alcohol services), how it is held and linked (held at a national level by Public Health Scotland in a secure setting, and linked using a national identifier), how researchers go about accessing this (talking through how researchers become accredited to use data and how they go about accessing data), and some examples of how it has been used (providing examples relating to drug research, including Tweed’s (2022) work on premature mortality in people affected by co-occurring homelessness, justice involvement, opioid dependence and psychosis [16], and Marryat’s (2023) Opioid in Pregnancy study [1]). Participants were able to ask questions about these processes before the group moved onto a more general discussion.

The discussion was guided by a Topic Guide (Supplementary File 1) and led by the focus group leader. Incorporated into this was a ranking exercise, where participants were asked to decide as a group how they felt about the use of different types of data, using a visual traffic light system, whereby red = did not want these data used/shared, yellow = ambivalent/unsure, and green = happy to share these data. Power dynamics were mitigated by asking participants to make independent decisions about where to place each type of data. After making their selections, we asked participants to explain their choice and used prompts to explore the underlying reasoning in greater depth. Focus groups lasted around 90 minutes and were recorded using an encrypted digital recording device, however, due to the noise levels in the settings, multiple note-takers were also employed to capture data as a back up. Additionally, a conference illustrator was employed to capture data visually, providing instant feedback to participants. Recordings were transcribed by a professional transcription service, with team members (LM and SR) checking the transcripts for accuracy. Identifiable information was removed from transcripts and a pseudonym was allocated to each participant. The non-identifiable transcripts were uploaded into NVivo v15 for data processing and analysis.

### Power Dynamics

The team were very aware that power dynamics were likely in this this situation, and that they had the potential to disrupt the project if not handled carefully [17]. Although it is not possible to completely remove such power dynamics, the team took steps to reduce these power dynamics as much as possible. These included asking organisation staff and volunteers to tell people about the project first (which may be easier to refuse engagement); wearing casual clothing rather than smart work dress to focus groups with participants; speaking to participants informally over coffee first; the team having lunch with participants each time.

### Analysis

A team approach was taken to analysis, with LM, CB, SG and SR carrying out thematic analysis, as guided by Braun and Clarke [18]. Working together, the team discussed and coded in a line-by-line approach, before revisiting the codes and data to create broader codes. This enhanced rigour and trustworthiness. These codes were then grouped into five broader themes, reflecting the critical factors impacting on people who use drugs views of their routinely-collected data being used by researchers.

## Results

Twenty-three participants took part in the two deliberative focus groups (Group 1 = n.12; group 2 = n.11). No data were recorded on the characteristics of participants, as the project was PIE in nature, however, it should be noted that both groups were heterogeneous in terms of gender and age.

Five themes were identified: awareness and transparency; consent and control; purpose, benefit and accountability, data quality, completeness and representation; and fear and mistrust. We will now present each of these in turn.

### Awareness and Transparency

Many participants reported that they had never considered whether and how their data were used in research. Others had some awareness that data were used in research, but did not have any detailed knowledge of this. There was a general feeling that people *should* know how their data were being used. Surprise was expressed as to which data were unable to be shared, for example, being unable to link child and father data together in Scotland (whilst mother-child data are able to be linked using a mother-child linkage key):

*“Of course, if it works for the mothers, then why not the dads. If there’s that information is there for the mother…kinda dads play an equal part. Ken what I mean, so why?” (Male 5)*

By contrast, the other group were less keen to link father and child data, citing previous negative experiences with midwives and social workers as colouring their views. Concerns were also raised that if people were more aware that their data were being shared, they might restrict what they say, with one participant suggesting that people might not attend services at all if they knew what they said would be shared:

*“That stops the individual going into services knowing that their information should be shared. It does it stops them” (Male 7).*

Another view was that data was ‘just a number’, and that participants did not feel strongly either way about that data being shared with researchers.

Awareness and transparency stemmed to what was found in research as well. Although there was a general consensus in favour of knowing what had been done with people’s data in research, there were differences of opinions in terms of how that should be delivered. One view favoured in-person delivery because that allowed researchers to be challenged on what they had done, ensuring those with literacy problems or who need additional support in understanding findings also have access to hearing about the research, and researchers appearing more trustworthy in-person:

*“You get a vibe offa someone…ken what I mean? Over the phone you can lie, you can tell them whatever the fuck they want tae hear. When you’re face to face it’s harder.” (Male 1)*

Discussions also considered that this may be challenging to deliver at scale. Another suggestion was that websites could be used to deliver information about research to a far wider group of people. Whichever way was used, there was a clear feeling that researchers needed to ensure that messaging was clear and simple:

*“Get straight to the point: this is what we’re doing, this is how it’s used, this is what we’re trying to influence” (Male 6).*

Transparency around all aspects of the data process was seen as critical, with a possibility for data sharing and use being seen as potentially secretive and exploitative for people who already feel heavily stigmatised and marginalised by service providers. An open line of feedback around how data are used was seen as instrumental in building trust between the research community and people who use drugs.

### Consent, Control and Autonomy

The surprise that data were being used without people’s knowledge naturally led to conversations around consent. Participants were generally unaware that routinely-collected data are unconsented in Scotland, with the exception of GP data. There was a strong view that all data should be consented, for example:

*“You would kind of feel violated if you opened up and shared something, for example like views, and then then later on find out that the even if it is anonymous, the information is later being shared with other people” (Female 2).*

It was felt that this would be a short exercise to collect consent at the start of an appointment. By contrast, another view was that the data would not be useful if consent was gathered as too few people would give their consent. Discussion was often tempered by thoughts around what data were being used for, stemming back to who has control over uses for these data:

*Male 4: Would it not just be that if everybody, for example, said no, you would never help make improvements.*

*Female 2: Well that’s true*

*Female 1: You wouldn’t get…the population, or the number of, say for education, different needs. If everyone kept saying no, and for whatever reason – of course, it’s personal, you know, they’ve maybe had a horrific time previously in services, so they are very, more declined to give information, and that’s classic…but I can’t, like, in a sense that, like you say, it is important because change will not happen unless you’ve got statistics. It can’t at least be reported, because I’m not saying things, that it all does work because there’s a lot out there and there’s a lot more still needing done, but people continue to get [in] the research. If people don’t share it then you wouldn’t get anything.*

*Male 6: But sometimes they share it for the wrong reasons and the bad stuff.*

*Female 1: Yes. Well, aye, and I have seen that. So. So I’m totally split with that because I have seen it being used for so many wrong reasons and it’s impacted on families and children, but the wrong…the wrong way…so yeah, totally. Totally. Yeah, yeah.*

Questions were also raised by participants about who should provide consent around death data e.g. should families provide consent for these specific data to be used. This was clearly a sensitive topic with strong feelings on both sides: for some reductions in drug-related and other deaths were an important reason to share and use these data, as was a feeling that you could no longer provide your consent. By contrast others thought that data around deaths should not be shared due to its sensitivity.

There were mixed views on sharing of data within and between services. One group argued that data should be used and shared to reduce burden and limit traumatisation of individuals, particularly where this was specifically asked for by individuals. Indeed, examples were provided where people wanted their data shared, but this was not acted on:

*“I do see the benefits of data being shared, however, not a lot of services are doing… and there’s not enough, not agency working. So very classic cases where families have ended up needing social work intervention and education aren’t talking and the data isn’t getting shared for the right reasons.” (Female 2)*

There was also a view that individuals lacked control as to whether their data were shared within services. There was a feeling that services would use the data even where consent was not given. Coercion within services was also apparent, with suggestions being made by service providers that if you have nothing to hide, then why would you not share your data? For others, though, there was a fear that data could be used to the individual’s detriment. This control over the purpose for using data, whether by services or research, was a recurrent theme.

### Stigma, Fear, and Mistrust

Stigma, fear and mistrust were central to many of the discussions held with the group. Concerns around how data were used often stemmed from prior experience of stigma and negative repercussions of data sharing within and between services. Many examples were shared of stigmatisation by services, with people feeling that they were treated as *“second class citizens”*, and that even within drug and alcohol services, participants felt that some people were judgemental and *“looking down their noses at you”*. Negative experiences with services were commonplace: as one participant explained *“everyone has been a victim of being lied to”*, and stigma coloured all conversations: *“we’re used to stigma around everything…waiting times…everything”*. This was frequently cited as a reason to worry about what data they provided to services and, in turn, what data were available to researchers.

Particular fears were raised about the impact of reporting data on child removal. For some, this meant not attending services at all, or limiting the information provided to those services:

*And that’s why people dinae want to go to addiction services or talk about addiction problems because they’re scared. Social work’s going to get involved…(Female 1)*

*I’ve got two young kids at home and for for so long, I was just putting that mask on because, sorry, because I felt that was what I had to do [coughing]. I know what can happen. (Male 5)*

### Purpose and Impact

When data were used for good there were generally high levels of support for data to be used in research. Use for ‘good’ was defined as improving experiences and services for individuals, for lobbying government to change policies, as well as improving outcomes for future generations:

*“But yeh, I just think anything that’s going to improve anything or for anyone, any service, any individual.” (Female 1)*

*“Because research into anything educational can only benefit your grandkids in the future, for your kids future, and your kid..kids future. So I’ve no got an issue with them researching mine.” (Male 7)*

Participants reflected on services and support that they felt they had not received, or which had been poor, particularly when it came to education. This motivated people to share their data to stop history repeating itself.

There were also concerns raised about what data might be used for, including the selling of information, and the use of data against individuals. Much of the discussion was centred on use of data for ‘the wrong reasons’, however this proved difficult to define. Concerns focussed on misuse of data by services, but again, discussion of data sharing with other services and external agencies vs. researchers was often difficult to disentangle. Data security was raised as an issue here, with fears around identity fraud reported.

### Data Quality, Completeness, and Representation

This theme was based on the fear of reprisal mentioned in the previous theme, and centred on a core concern about whether routinely-collected data accurately and fairly represented people’s experiences within services. One of the key aspects highlighted within this was missing data. Two reasons were provided for this: firstly that people were missing from data due to not being able to access services when they tried, for example not being able to get a psychiatrist or GP appointment; and secondly, avoiding engaging with services due to previous experiences, as mentioned earlier. For example, one participant reported that their *“data would be inconclusive because I didnae get the right treatment so I don’t go to the doctors for that reason…” (Male 1).* This was often wrapped up in experiences of stigma as a person who uses drugs, with examples given of feeling that a GP perceived the person to be seeking to gain access to opioids by falsifying symptoms. This was linked to the accuracy of data. Again, whilst this was sometimes thought to be due to mis-recording of data by services, with what people say getting “twisted”, it was also highlighted that the information provided by participants was sometimes self-selected. For example “*I was actually really struggling, but she couldn’t come out and visit, so, and I was telling her that everything was alright so she didn’t come out and get involved so...” (Female 1).* Participants highlighted that data would therefore be missing or incomplete and questioned the value for this population in some cases.

## Discussion

This public involvement and engagement study aimed to ascertain the views of sharing data for research by people who use drugs in order to gauge the extent to which they supported such data use. Overall, results highlighted the lack of awareness about what happens to people’s data and how it is used for research, in line with findings from other groups [4]. On learning more about how routinely-collected data are used in research, feelings were mixed, with concerns raised over a lack of control over how data are used, but with high levels of support where data are used to benefit the community. This largely aligns with findings from other populations, where a conditional social licence is provided [4]. Where findings were strikingly different from other populations involved in similar studies was a lack of trust of health services. Whereas other populations have reported a hierarchy of trust, with health services at the top, followed by researchers and then commercial organisations at the bottom [4], for people who use drugs, there was a severe lack of overall trust in health services. This resulted in greater trust in researchers, and less trust in health services. Commercial organisations interests were largely ignored, though the group were not explicitly asked about these views. Much of the distrust in services stemmed from the structural inequalities experienced by people who use drugs. They described widespread experiences of stigma across a range of services, many of which were directly there to support this particular population, as has been extensively documented [8]. Experiences of traumatisation through repeated retelling of stories, and fear of reprisal from services when being honest about life challenges, added to this sense of fear and mistrust of services. Whilst there was no sense that this distrust extended to researchers, the impact of this on data used by researchers remains, as data quality was highlighted as being limited in some instances. We can also not underestimate the potential for the power imbalance between the team carrying out this study, and participants, to have affected the discussions, despite the team’s best efforts to reduce this [17]: if staff within the recovery service had asked participants how they felt about researchers using their data, answers might have been different.

Power dynamics at play between service providers and people who use drugs more generally left participants feeling a lack of control over how their data were used. This was particularly the case when consent within services to share data had been denied by participants, yet it was reported that this data was shared without permission. This lack of trust was reiterated several times by participants, with a feeling that their views and opinions were worthless. This ‘epistemic injustice’ has been increasingly recognised among marginalised groups, such as people who use drugs, with their views being seen as less credible or trustworthy [19]. Empowering the voices of these individuals was therefore critical to this project. Indeed, the inclusion of a conference illustrator to provide instant feedback that the groups’ voices were being heard and noted, was commented on by participants, and the team would highly recommend this to other research and PIE teams carrying out similar work. Additionally, a short film was created by a separate group of people who use drugs, to put these viewpoints in participants own voices and images [20]. Previous research has highlighted the important role that creative methods can play when working with vulnerable people, through providing diverse ways to understand topics, and enabling participants to share experiences, concerns, and challenges more comfortably with researchers through creating a safe environment [21].

In light of these views, the research community, should take heart that a social licence exists for use of routinely-collected data, where this is being used to benefit the community of people who use drugs and their families. Researchers and data custodians should not rest on their laurels, however, as the social licence can be withdrawn, to often devastating effects [3]. To ensure the social licence to use routinely-collected data of people who use drugs in research continues, it is recommended that researchers working with these data following the principles set out in the Public Engagement in Data Research Initiative (PEDRI) Good Practice Standards [22]. These include two-way communication with people whose data are being used to enable better understanding of each others’ views and to empower people whose data we are using to contribute effectively to the conversation about how their data are used. Other principles also reflect the views held in this group, including ensuring transparency around data use and ensuring mutual benefit [22]. Through endeavouring to fulfil these principles, researchers will go some way to addressing previous epistemic injustice. It is acknowledged that this often requires a different skillset to that which analysts working with routinely-collected data are used to, and that this can be intimidating [23]. The participants in this study felt strongly however that this level of transparency was required to enable the social licence to be fulfilled. With this in mind, funders need to enable these types of activities, particularly for early career researchers, who may not hold large grants to fund initial relationship building work.

### Strengths and Limitations

This study was successful in engaging people who use, or have used, drugs in discussions around the use of their data, which have previously been neglected in studies examining the social licence for routinely-collected data use in research. Analysis of data was carried out by multiple team members, whilst findings were fed back to participants and others in the recovery organisation to obtain feedback and enable verification of results. Limitations included that, due to resource, focus groups were carried out in one recovery organisation across two different areas within South-East Scotland. Views may be different across different geographical areas, due to different health services and systems, although a recent review of stigma in health services towards people who use drugs found this to be universal [8]. Intersectionality may also affect findings: whilst we captured reasonable diversity in terms of gender and age in our sample, we did not capture people from ethnic minority communities well, or those further marginalised through imprisonment. As this study was PIE rather than research, limited demographic data were collected.

## Conclusions

This paper provided broad support for a social licence to use routinely-collected data, by people who use drugs. This was conditional on research being conducted to ‘do good’, namely improving the services and experiences of people who use drugs and their families. Despite a lack of awareness that data are currently used in research, the participants in our study were enthusiastic about hearing more about research using their data, and demanded transparency and accountability in relation to this from researchers. The inclusion of the views of people who use drugs in research design is important, not only to begin to address epistemic injustice and mistrust within this group, but also to improve the quality and diversity of research being delivered, ensuring that this truly addresses the needs of the community in question.

## Supplementary Files

Supplementary File 1

## Rights Retention Statement

For the purpose of open access, the author(s) has applied a Creative Commons Attribution (CC BY) licence to any Author Accepted Manuscript version arising from this submission.

## CREDIT Statement

Conceptualisation: LM, HB, SG, AM, ST and CBD. Methodology: LM, HB, SG, AM, SR, ST and CBD. Formal analysis: LM, CBD, SG, SR. Writing – original draft: LM. Writing – reviewing and editing: LM, HB, SG, AM, SR, ST and CBD. Funding acquisition: LM, HB, SG, AM, ST and CBD.

## Data Access Statement

This was a public engagement project with findings suitable for publication. No research data was collected as part of this and data collected as part of the public engagement project are not available for sharing beyond the research team.
