# Supplementary material for: “If it’s gonnae make things better then I don’t have any issue”: Perspectives of People Who Use Drugs on the Use of Routinely-Collected Data for Research
Source: Int J Popul Data Sci. 2026 Jun 30;6(1):3428. doi: 10.23889/ijpds.v11i1.3428 (PMC13359100; doi:10.23889/ijpds.v11i1.3428)
Supplement: Supplementary File 1 [file ijpds-11-3428-s001.pdf]

Supplementary File 1: Deliberative focus group Topic Guide

Topic Guide Focus Group (1-2 hours)

My data: an animated film, co-produced with people who use substances

Start of focus group

- Introduction between researchers and participants
- Participant Information Sheet: researcher to discuss the form with the participants and answer any questions
- Reinforce that we are just discussing health-related data and not anything wider than that (e.g. criminal record, risk assessment)
- Reinforce don't want to know anything about individual health conditions (e.g. chronic conditions, BBV) – we just want to know what you think about the routinely-collected data and how it is used
- Consent – record verbal consent

Focus Group: key topics for discussion

- Discussion around data – what are routinely-collected information (e.g. hospital records, prescription data), how are data currently stored and linked
- The research team will share examples of the current use of data/information and what is classified as routinely-collected data/information (the team will select visual materials with examples of data/information use to share with the participants (e.g. pictures and short film clips)
- Discussion around routinely-collected data/information and health services
    - What do you think happens with the data/information related to your previous drug use/ drug use history when shared with your GP/pharmacy?
- What is anonymous data/information for you? (e.g. drug-related deaths)
- How do you feel about how your data/information is shared/used?
- Do you feel comfortable with how your routinely-collected data/information is used? Are there any data/information that you would feel/ not feel comfortable being used? Does the purpose of data/information collection make a difference to how you feel? How do you feel about the use of your data/information? Is there anything researchers could do to make you more likely to want to share your data for research? Is there anything that stops you wanting your data used in research?

  Schedule activity: The team will use three big A3 sheets of paper (red, amber, green paper) with a person printed on the card, with pictures/names of different small cards which they can put where they are comfortable or not in terms of data being used. The team will use this process for service use, for research, and for commercial use.
- How would you like to be informed about the research results from data/information?
- Discussion around their routinely-collected data/information and their rights.

Close of focus group

- Stop recording
- Thank participants for their time
- Explain the next steps and timescale (14 days to withdraw data)
- Ask if they would like to be informed of the findings
- Ask if they would like to be part of the creative workshops
